# The excess mortality risk of diabetes associated with functional decline in older adults: Results from a 7-year follow-up of a nationwide cohort in Taiwan

**DOI:** 10.1186/1471-2458-11-953

**Published:** 2011-12-23

**Authors:** Chia-Lin Li, Hsing-Yi Chang, Yea-Ing L Shyu

**Affiliations:** 1 Department of Health Care Management, Chang Gung University, 259 Wen-Hwa 1st Road, Kwei-Shan, Tao-Yuan 333, Taiwan; 2 Division of Health Policy Research and Development, Institute of Population Health Sciences, National Health Research Institutes, 35 Keyan Road, Zhunan Town, Maoli County, Taiwan; 3 School of Nursing, Chang Gung University, 259 Wen-Hwa 1st Road, Kwei-Shan, Tao-Yuan 333, Taiwan

## Abstract

**Background:**

Diabetes is associated with an increased risk of functional decline in older adults. Few studies have investigated the contribution of functional decline to excess mortality risk in older people with diabetes. The aim of this study was to examine how diabetes in combination with different levels of functional decline affects 7-year mortality in older adults.

**Methods:**

We analyzed data from a nationally representative sample of people aged 65 years and over, participating in the 2001 National Health Interview Survey in Taiwan. A total of 1873 participants were followed through 2002-2008, of whom 286 (15.3%) had a history of diabetes confirmed by a medical professional. Participants were divided into three functional status groups: (1) high functioning-no limitations involving activities of daily living (ADLs), instrumental activities of daily living (IADLs), or physical functioning; (2) low functioning-limitations in one or more ADLs; (3) middle functioning-all participants in between groups 1 and 2.

**Results:**

The crude mortality rate was 52.7 per 1,000 person-years in those with diabetes and 34.1 per 1,000 person-years in those without diabetes. After adjustment for other factors, diabetes alone was not associated with an increased mortality risk in those with high functioning. However, diabetes alone had a hazard ratio (HR) for mortality of 1.90 (95%CI = [1.02-3.53]) in those with middle functioning and 3.67 (95%CI = [1.55-8.69]) in those with low functioning. The presence of diabetes and one or more other chronic conditions was associated with a HR for mortality of 2.46 (95%CI = [1.61-3.77]) in those with middle functioning and 4.03 (95%CI = [2.31-7.03]) in those with low functioning.

**Conclusions:**

Our results indicate that diabetes is not associated with increased mortality in those with high functioning. There was a gradient effect of functional decline on mortality in individuals with diabetes. Additionally, among participants with other chronic conditions, functional decline was associated with a greater burden of mortality in older adults with diabetes. These findings highlight the critical importance of the prevention of cardiovascular disease morbidity and the maintenance of functional abilities in order to reduce mortality risk in older adults with diabetes.

## Background

Most studies of health outcomes in older adult populations have consistently demonstrated that diabetes mellitus is associated with a high prevalence of functional impairment or disability [1-4]. However, past studies have not explored whether this functional decline contributes to the excess mortality risk associated with diabetes in older adults. Given the great diversity of functional decline in older adults with diabetes [5,6], there is increasing evidence to support the importance of recognizing functional status as a part of individualized care in older adults with diabetes. Assessment of the impact of diabetes on mortality combined with different levels of functioning may provide a more accurate prediction of mortality risk, which will assist health care providers in targeting interventions for those at highest risk of mortality.

Epidemiological data from a nationally representative sample of U.S. older adults, the National Health and Nutrition Examination Survey (NHANES 1999-2006), found that diabetes-related comorbidities such as cardiovascular disease may contribute to a greater burden of disability in those with diabetes [7]. Older adults with diabetes have more comorbidities, particularly those related to diabetes complications, which are associated with an increased risk of mortality [8]. Data from the U.S. Cardiovascular Health Study (CHS) has shown that in people diagnosed with diabetes at age 65 years and over, diabetes is associated with increased cardiovascular mortality at an earlier phase of the disease process [9]. In the Hoorn Study, Spijkerman et al. [10] demonstrated that among participants aged 50 to 75 years with a short diabetes duration, mortality risk could largely be attributed to cardiovascular risk factors. These findings indicate that comorbid cardiovascular disease is associated with both functional decline and mortality in older adults with diabetes. Despite this evidence, few studies have examined the interrelationship between comorbidities, functional decline and risk of mortality in older adults with diabetes.

The population in Taiwan is rapidly aging. Diabetes has been the 4th or 5th leading cause of death since 2002 and Chang et al. [11] showed that nearly 25% of individuals aged ≧ 65 years have diabetes in Taiwan. Our previous research has shown that people aged 65 and over with diabetes are more likely to use medical services [12]. The growing burden of this disease and its associated health care costs reinforce the importance of providing effective management for older adults with diabetes. Understanding the potential factors contributing to increased mortality in older people with diabetes could help in the development and implementation of strategies for reducing premature mortality in this population.

In view of these considerations, we analyzed data from a 7-year prospective study of a nationally representative sample of older adults aged 65 years and over in Taiwan. The main aim of this study was to examine how diabetes in combination with different levels of functional decline affects mortality in older adults, particularly by stratifying our participants by the presence or absence of other chronic conditions. We classified participants into three groups based on self-reported functional status. This functional classification schema is a modified version of several well-established classification schemes [5]. We also aimed to describe variability in baseline functioning and associated factors in older adults with diabetes.

## Methods

### Study population

This was a prospective study involving participants in the National Health Interview Survey (NHIS) in Taiwan, 2001. The multistage stratified sampling design of the NHIS has been described previously in detail [13]. The original sample comprised 22,121 participants (response rate; 94.2%), including 2,064 individuals aged ≧ 65 years. The data has been released to the public http://nhis.nhri.org.tw. The survey has obtained the ethical approval from Institutional Review Board of the National Health Research Institutes. All study participants signed the informed consent. Of these potential participants, we excluded 18 who had incomplete data for history of self-reported diagnosed diabetes or age at diagnosis, 53 who had incomplete data for history of heart disease, hypertension, and stroke, 99 who had incomplete data for self-reported functioning, and 21 who had died by the end of 2001. This resulted in 1873 eligible participants for analysis. The study cohort was followed until December 31, 2008 through linkage via a personal identification number to the computerized data files of the National Register of Deaths.

### Demographic characteristics, health status, and comorbid conditions

Trained interviewers interviewed all participants using standard questionnaires and collected baseline data including age, sex, years of education, marital status, and smoking status. We also assessed factors considered to be associated with mortality in people with diabetes including the age at diagnosis, use of insulin, and the presence of other chronic conditions, such as a history of heart disease, hypertension, and stroke. For each disease (including diabetes), individuals were asked whether the diagnosis had been confirmed by a medical professional.

### Functional status groups

This functional classification schema is a modified version of several well-established classification schemes [5]. Participants were asked to report their ability to perform functioning tasks consisting of personal care tasks (six activities of daily living (ADLs)) including bathing, dressing, eating, using the toilet, getting in or out of bed, and walking across a small room; assessment of complex social functioning (instrumental activities of daily living (IADLs)) including the ability to perform housework such as preparing meals and sweeping; and physical functioning tasks including two mobility tasks (walking several blocks, climbing one flight of stairs), and one strength task (lifting or carrying groceries). Participants were asked whether they could perform ADL and IADL activities without help, with a little help, with considerable help, or were unable to perform the activities. Limitation in each ADL and IADL task was dichotomized as needing no help or a little help versus needing considerable help or being unable to perform the activity. Participants were asked whether they could perform physical functioning tasks with no limitation, with a little limitation, or with a lot of limitation. Participants were divided into three groups based on functional status: (1) high functioning group, defined as reporting no limitations involving ADLs, IADLs, or physical functioning; (2) low functioning group, defined as reporting limitations in one or more ADLs, i.e., including reporting limitations in ADLs alone, or reporting limitations in ADLs combined with limitations in IADLs and/or physical functioning; (3) middle functioning group, defined as all participants in between groups 1 and 2, i.e., those participants reporting no limitations in ADLs, but reporting limitations in IADLs and/or physical functioning.

### Statistical analysis

We used the Student's *t*-test and Pearson's Chi-Square test to compare the characteristics of those with and without diabetes. We used the Pearson's Chi-Square test to compare categorical variables and one-way analysis of variance to compare continuous variables between functional status groups at baseline. Cox proportional hazards models were used to estimate the associations between baseline characteristics and mortality. Adjusted hazard ratios (HR) and 95% confidence intervals (95% CI) for mortality were estimated. All analyses were conducted using SAS version 9.1 (SAS Institute, Cary, NC) and SPSS version 14.0 (SPSS, Chicago, Ill., USA).

## Results

The baseline characteristics of older adults with and without a history of diagnosed diabetes are presented in Table 1. Among adults aged ≧ 65 years, the prevalence of diagnosed diabetes was 15.3%. In those with diabetes, 32.5% had high functioning compared to 50.5% without diabetes. The prevalence of low functioning was 13.6% in those with diabetes and 6.6% in those without diabetes. Older adults with diabetes were more likely to have middle or low functioning and have other chronic comorbidities such as heart disease, hypertension, and stroke.

**Table 1:** Baseline participant characteristics by history of diagnosed diabetes

	History of diagnosed diabetes			P-value*
		
	Overall	No	Yes	
N	1873	1587	286	

Age (years; mean ± SE)	73.0 ± 0.14	73.0 ± 0.15	73.1 ± 0.36	0.943

Sex (% female)	48.7	48.7	48.6	0.973

Education (years; mean ± SE)	4.9 ± 0.11	4.9 ± 0.12	5.1 ± 0.30	0.535

Marital status (%married or living with partner)	66.8	67.2	64.3	0.338

Smoking status (%current)	19.4	19.7	17.8	0.454

Functional status (%)				

High	47.7	50.5	32.5	< 0.001

Middle	44.6	43.0	53.8	

Low	7.6	6.6	13.6	

Heart disease (% yes)	23.4	21.6	32.9	< 0.001

Hypertension (% yes)	39.6	37.3	52.3	< 0.001

Stroke (% yes)	7.6	6.4	14.4	< 0.001

Number of other conditions^† ^(%)				

0	49.6	52.1	35.3	< 0.001

1	32.8	32.4	34.5	

≥ 2	17.7	15.4	30.2	

Duration of diabetes (years; mean ± SE)			10.0 ± 0.55	-

Use Insulin (% yes)			19.7	-

*Continuous variables were compared using the Student's *t*-test and shown as the mean ± standard error (SE). Categorical variables were compared using Pearson's Chi-square test and shown as percentages.

^†^Other conditions include heart disease, hypertension, and stroke.

Table 2 presents the baseline characteristics of older adults by different levels of functioning for participants with or without diabetes. The distribution of baseline age, sex, years of education, marital status, current smoking status, and number of other chronic comorbidities were significantly different between functioning groups, in both participants with or without diabetes. In participants with diabetes, those with middle or low functioning had a borderline significant longer duration of diabetes.

**Table 2:** Baseline health-related characteristics by functional status for participants with and without diabetes

	No history of diagnosed diabetes (N = 1587)				History of diagnosed diabetes (N = 286)			
				
	Functional Status				Functional Status			
	
	High	Middle	Low	P-value*	High	Middle	Low	P-value*
N	801	682	104		93	154	39	

Age (years; mean ± SE)	71.3 ± 0.17	74.2 ± 0.22	79.1 ± 0.71	< 0.001	71.4 ± 0.51	72.8 ± 0.44	78.3 ± 1.27	< 0.001

Sex (% female)	37.8	60.4	55.8	< 0.001	39.8	48.1	71.8	0.003

Education (years; mean ± SE)	5.6 ± 0.17	4.2 ± 0.18	3.8 ± 0.47	< 0.001	5.9 ± 0.53	5.1 ± 0.42	2.8 ± 0.62	0.006

Marital status (%married or living with partner)	72.8	62.5	55.8	< 0.001	69.9	66.9	41.0	0.004

Smoking status (%current)	25.1	15.1	8.7	< 0.001	25.8	15.6	7.7	0.026

Heart disease (% yes)	16.8	26.2	28.8	< 0.001	23.7	36.4	41.0	0.060

Hypertension (% yes)	32.7	40.6	52.0	< 0.001	46.2	55.6	53.8	0.356

Stroke (% yes)	2.4	5.6	42.7	< 0.001	2.2	15.1	41.0	< 0.001

Number of other conditions^† ^(%)								

0	59.3	47.1	29.7	< 0.001	47.3	30.4	25.6	0.007

1	30.3	35.5	28.7		34.1	36.5	28.2	

≥2	10.4	17.3	41.6		18.7	33.1	46.2	

Duration of diabetes (years; mean ± SE)	-	-	-	-	8.9 ± 0.98	10.0 ± 0.72	12.8 ± 1.68	0.085

Use insulin (% yes)	-	-	-	-	13.0	22.2	25.6	0.131

*Continuous variables were compared using one-way analysis of variance and shown as the mean ± standard error (SE). Categorical variables were compared using Pearson's Chi-square test and shown as percentages.

^†^Other conditions include heart disease, hypertension, and stroke.

Table 3 presents the number of deaths, person-years, mortality rate, and adjusted HR and 95% CIs. There were 88 deaths in the 286 persons with diabetes, giving a crude mortality rate of 52.7 per 1,000 person-years. In those without diabetes, there were 340 deaths out of a total of 1587 persons giving a crude mortality rate of 34.1 per 1,000 person-years. After adjustment for age, sex, education, marital status, current smoking, heart disease, hypertension, and stroke, the HR for mortality for older adults with diabetes compared to those without diabetes was 1.28 (95% CI = [1.00-1.64]), and the HRs for mortality for older adults with middle functioning and low functioning compared to those with high functioning were 1.41 (95% CI = [1.12-1.77]) and 3.28 (95% CI = [2.32-4.63]), respectively.

**Table 3:** Adjusted Hazard Ratios (HR) and 95% Confidence Intervals (95% CI) for mortality

	Total	History of diagnosed diabetes		Functional status		
		
		No	Yes	High	Middle	Low
N	1873	1587	286	894	836	143

Deaths	428	340	88	140	204	84

Person years	11644.0	9974.1	1669.9	5848.1	5147.9	648.1

Mortality Rate (per 1,000 person-years)	36.8	34.1	52.7	23.9	39.6	129.6

Model*: HR (95% CI)		Reference	1.54 (1.22-1.95)	-	-	-

Model^†^: HR (95% CI)		Reference	1.43 (1.13-1.83)	-	-	-

Model*: HR (95% CI)		-	-	Reference	1.48 (1.19-1.86)	3.60 (2.66-4.88)

Model^†^: HR (95% CI)		-	-	Reference	1.43 (1.14-1.80)	3.44 (2.44-4.84)

Model^†^: HR (95% CI)		Reference	1.28 (1.00-1.64)	Reference	1.41 (1.12-1.77)	3.28 (2.32-4.63)

*Adjusted for age and sex.

^†^Adjusted for age, sex, education, marital status, current smoking, heart disease, hypertension, and stroke.

Table 4 presents the joint effects of diabetes and functioning on mortality with or without the presence of other chronic conditions, such as heart disease, hypertension, and stroke. After adjustment for age, sex, education, marital status, and current smoking, compared to those with high functioning, no diabetes and no other chronic conditions (reference group), older persons with diabetes combined with one or more other chronic conditions had a borderline significant increased mortality risk (HR = 1.84; 95% CI = [0.97-3.48]). Compared to the reference group, older persons with diabetes and no other chronic conditions had a HR for mortality of 1.90 (95% CI = [1.02-3.53]) if they had middle functioning and a HR of 3.67 for mortality (95% CI = [1.55-8.69]) if they had low functioning. In older persons with diabetes and one or more other chronic conditions, the HR for mortality was 2.46 (95% CI = [1.61-3.77]) in those with middle functioning and 4.03 (95% CI = [2.31-7.03]) in those with low functioning.

**Table 4:** Adjusted* Hazard Ratios (HR) and 95% Confidence Intervals (95% CI) for mortality by diabetes and different levels of functioning, after stratifying for other chronic conditions

	No diabetes		Diabetes	
	
	HR (95% CI)	P-value	HR (95% CI)	P-value
High functioning				

No other conditions	Reference		1.33 (0.64-2.78)	0.444

One or more other conditions	1.20 (0.83-1.72)	0.328	1.84 (0.97-3.48)	0.063

Middle functioning				

No other conditions	1.57 (1.12-2.21)	0.009	1.90 (1.02-3.53)	0.042

One or more other conditions	1.50 (1.07-2.10)	0.018	2.46 (1.61-3.77)	< 0.001

Low functioning				

No other conditions	3.31 (1.90-5.78)	< 0.001	3.67 (1.55-8.69)	0.003

One or more other conditions	4.60 (3.08-6.87)	< 0.001	4.03 (2.31-7.03)	< 0.001

*Adjusted for age, sex, education, marital status, and current smoking.

^†^Other conditions include heart disease, hypertension, and stroke.

## Discussion

Our joint analysis results indicate that there is no excess mortality risk associated with diabetes in those with high functioning. However, in older adults who had diabetes combined with functional decline, the excess mortality risk was substantially increased. Importantly, we also found that among participants with other chronic conditions, diabetes combined with functional decline was associated with a greater burden of mortality in older adults. In addition to maintaining functional status, our findings underscore the critical importance of the prevention of cardiovascular disease morbidity which could lead to improved survival in older adults with diabetes.

To our knowledge, this study is the first to provide evidence of a gradient response between functional impairment and mortality in older persons with diabetes. After adjustment for age, sex, education, marital status, current smoking, heart disease, hypertension, and stroke, the relative risk of mortality associated with diabetes was 1.43 (95% CI = [1.13-1.83]) (Table 3). This estimate is consistent with the majority of studies on general population samples that have reported an increased mortality associated with diabetes in older adults of less than 1.5 fold [14-17]. Our study differs from most previous studies in that we further explored the effects of combinations of functional decline which more accurately reflect the true picture in the heterogeneous population of older people with diabetes. It is notable that among individuals aged 65 and older in Taiwan, only 32.5% of those with diabetes reported no functional problems (high functioning group). Of these participants, only 47.3% reported no other chronic conditions and there was no increased mortality risk over a 7-year follow-up in this group. However, 52.7% of those with diabetes and no functional limitations had one or more other chronic conditions and also demonstrated a borderline significant increased risk of mortality. This study found that 53.8% and 13.6% of those with diabetes reported middle and low functioning, respectively. These two groups were more likely to have other chronic conditions and their mortality risk was substantially increased.

As cardiovascular diseases such as hypertension, heart disease, and stroke are the most important contributors to mortality in older adults with diabetes [9,10,18,19], we further stratified our participants by the presence or absence of these chronic conditions. Although functional decline is one of the most important contributors to mortality among older adults, few studies have demonstrated a dose-response relationship between functional decline and mortality in older adults with diabetes. After adjustment for age, sex, education, marital status, and current smoking, our results extend previous studies by demonstrating a gradient effect of functional decline on mortality in individuals with diabetes, with or without the presence of other chronic conditions. In addition, among participants with other chronic conditions, functional decline was associated with a greater burden of mortality in older adults with diabetes (Table 4). These results indicate that the excess mortality risk associated with functional decline may be partially attributed to the presence of comorbidities among older adults with diabetes. Our results have important practical implications in that they indicate that single-disease orientated diabetes care may not be enough. Rather, additional care that focuses on the prevention or the early detection and control of diabetes-related comorbidities should be emphasized. This broader approach is needed to satisfy the complex health care needs of older adults with diabetes. Our results show that the magnitude of the association between functional decline and mortality is greater than that for diabetes or comorbidities, and therefore they indicate that functional decline is more strongly related to death. Further investigations are needed to examine the prospective associations between comorbidities and functional decline in older adults with diabetes to provide greater insight into the interdependent pathways between comorbidities, functional decline and mortality. Intervention studies are also required to confirm whether older persons with diabetes would benefit from interventions aimed at preventing comorbidities, and whether this reduced comorbidity load leads to better functioning and survival.

It is notable that for the same level of decline in functional status, individuals with diabetes were younger than those without diabetes (Table 2). This supports previous research indicating that individuals with diabetes develop the decline in functioning associated with aging earlier than those without diabetes [17,20,21]. In a cross-sectional analysis of 3075 well-functioning older adults aged 70-79 [1], poor glycemic control was associated with risk of early functional decline in individuals with diabetes. Recent research based on UKPDS (United Kingdom Prospective Diabetes Study) risk models further demonstrate that the presence of functional impairments may diminish the benefits of achieving intensive glucose control [22]. Among older adults with diabetes, prior research suggests that an association exists between hyperglycemia and frailty, muscle weakness, and poor muscle quality [23,24]. Moreover, poor muscle quality in older adults with diabetes deteriorates further with longer diabetes duration and higher levels of HbA1C [25], which are associated with increased mortality [26]. The results from the Health, Aging and Body Composition Study [27] have suggested that poor peripheral nerve function partly explains worse physical performance and may be directly associated with physical performance in older adults with diabetes. Previous studies have documented that one or more vascular complications are associated with functional impairments in individuals with diabetes [28]. These could be possible mechanisms behind our findings of excess mortality associated with reduced functioning in individuals with diabetes.

In the present study, it is not clear why no excess mortality risk was associated with diabetes among high functioning participants. It is possible that maintenance of high functioning in individuals with diabetes results in greater independence and a greater likelihood of performing self-care activities associated with better diabetes control and better survival. It is also possible that better health status is contributing to the association between high functioning and no excess mortality in individuals with diabetes. In this study among participants with diabetes, people with high functioning were significantly more likely to be younger, more educated, male, married or living with a partner, and current smokers, compared to those with middle or low functioning (Table 2). They were less likely to have a history of stroke, and less likely to have one or more other chronic conditions. They also had a borderline significant shorter duration of diabetes. These results are consistent with past studies among older adults with diabetes that have reported associations between increasing functional decline, increasing age and comorbid cardiovascular diseases such as hypertension, heart disease, and stroke [3,4,7].

Our study has several limitations. First, we did not have clinical data on glycated hemoglobin, severity of diabetes symptoms, diabetes complications, cancer, and antidepressants. These factors can be associated with both mortality and functional status in people with diabetes, and therefore may have confounded our results. Second, we cannot exclude a possible effect of prevalent sub-clinical disease that might be associated with both impaired functioning and increased mortality in people with diabetes. Third, in this study, all participants were 65 years and above, which may have resulted in survival bias. As participants had to be 65 years and above at study entry to meet the study inclusion criteria, our sample of persons with diabetes could be biased towards those with better health status which has enabled them to survive into older age. Therefore, the association between functional decline and mortality may be underestimated. As we were still able to demonstrate a statistically significant association between functional status and mortality despite this potential underestimation, it is likely that the association between functional decline and risk of mortality among older adults with diabetes is substantial.

Despite these limitations, our study has several strengths including the analysis of a nationwide representative sample of the Taiwanese population through linkage of NHIS data and the National Register of Deaths. This study found that as many as 52.2% of people aged 65 and older in Taiwan reported functional decline (Table 1). It is notable that among those participants with low functioning status, 94.4% (135/143) reported limitations in all of the three functioning domains of ADLs, IADLs, and physical functioning, and only 5.6% (8/143) reported limitations in ADLs and physical functioning without limitations in IADLs. Thus, the majority of participants (94.4%) in the low functioning group actually had limitations in at least one task in each of the three different functioning domains. These findings have important implications for health care planning and resource allocation to support improved functioning in older adults.

Our results indicate that using a simple functional classification can identify older adults with diabetes who are at a higher risk of mortality in a relatively inexpensive manner in the absence of laboratory and clinical indicators. Our findings have important practical implications for developing intervention strategies to support the care of older adults with diabetes. Our findings suggest that healthcare providers could use this classification approach to stratify the care of older adults with diabetes according to functional status. Health care strategies for older adults with diabetes and high functioning could focus on the management of cardiovascular risk factors and the reduction of cardiovascular disease morbidity. In contrast, strategies for older adults with diabetes and poorer functioning could be aimed at improving function through early evaluation and management of underlying physical disorders and other chronic conditions.

## Conclusions

In summary, our results highlight that there is a gradient effect of functional decline on mortality in individuals with diabetes. Health care professionals should pay careful attention to functional status among older adults with diabetes. In addition, health promotion programs aimed at improving survival among older adults with diabetes should emphasize the prevention of cardiovascular disease morbidity.

## Competing interests

The authors declare that they have no competing interests.

## Authors' contributions

CLL initiated the study, carried out the data analysis, and drafted and revised the manuscript. HYC conducted the NHIS survey, reviewed the data, discussed the study, and revised the manuscript. YILS reviewed the data, discussed the study and provided valuable comments on the manuscript.

## Pre-publication history

The pre-publication history for this paper can be accessed here:

http://www.biomedcentral.com/1471-2458/11/953/prepub
